# Structure, composition and biological properties of fungal extracellular vesicles

**DOI:** 10.1093/femsml/uqab009

**Published:** 2021-06-24

**Authors:** Juliana Rizzo, Adam Taheraly, Guilhem Janbon

**Affiliations:** Unité Biologie des ARN des Pathogènes Fongiques, Département de Mycologie, Institut Pasteur, F-75015 Paris, France; Unité Biologie des ARN des Pathogènes Fongiques, Département de Mycologie, Institut Pasteur, F-75015 Paris, France; Ecole Doctorale BioSPC, Université de Paris, 75006 Paris, France; Unité Biologie des ARN des Pathogènes Fongiques, Département de Mycologie, Institut Pasteur, F-75015 Paris, France

**Keywords:** fungi, extracellular vesicles, pathogens, intercellular communication, virulence, interkingdom communication

## Abstract

Extracellular vesicles (EVs) are lipidic nanosized particles that deliver a highly complex molecular cargo between cells and organisms and may serve numerous functions in intercellular communication, thereby influencing the evolution of microbial communities. Their roles in infectious diseases have been studied for a long time, comprising viral, bacterial, parasitic and to a less extent, fungal infections. Over the last few years, fungal EVs have become an increasingly active research field. Nevertheless, the understanding of EV functions during fungal infections poses challenging points, comprising the genetics regulating EV release, the EV structural and compositional complexity, the heterogeneity of the EV populations and their impact on host-pathogen interactions. This review explores the state-of-the-art investigations on fungal EVs and how this fast-evolving field can impact the development of new tools to fight fungal infections.

## Teaser

The study of fungal extracellular vesicles is a fast evolving field which will impact the development of new tools to fight fungal infections in the future.

## INTRODUCTION

Extracellular vesicles (EVs) are membranous compartments that enclose diverse biomolecules and are released by virtually all living cells in the three domains of life (Gill, Catchpole and Forterre 2019). EVs isolated in different studies vary in size, cargo, density and morphology and originate either from the plasma membrane or the endocytic trafficking pathway (Mathieu *et al*. 2019). Extracellular vesicles have been suggested as universal communication players in the microbial world (Tsatsaronis *et al*. 2018). They were reported as circulating particles during the development of different human pathologies and infectious diseases (Schorey and Harding 2016; Szempruch *et al*. 2016; Hosseini-Beheshti and Grau 2018; Shah, Patel and Freedman 2018), as well as in different environmental niches, such as marine ecosystems (Biller *et al*. 2014). Three major EV subtypes have been studied and discussed by several groups: exosomes, microvesicles and apoptotic bodies. Exosomes (30–150 nm) are formed in the endosomal pathway through the inward budding of endosomal membranes during their maturation into multivesicular bodies (MVB) that bind to the plasma membrane and release the intraluminal vesicles (ILVs) through exocytosis (Gandham *et al*. 2020). Microvesicles (100–1000 nm) are formed by the outward budding of the plasma membrane, and apoptotic bodies are vesicles formed during apoptosis which are usually larger than 1000 nm (Gandham *et al*. 2020).

EVs were first described as ‘platelet dust’ by Peter Wolf in 1967, and for a long time, they were considered debris of damaged cells (Wolf 1967). However, over the past few decades, EVs were shown to be not simply inert cellular products but biologically active particles instead, regulating cell physiology, delivering virulence-associated molecules, mediating the long-distance transfer of genetic information, among other complex functions (Mathieu *et al*. 2019; Pegtel and Gould 2019). Extracellular vesicle release has been reported in various microorganisms, such as bacteria, archaea, protozoa, virus-infected cells and fungi and the impact of EV production during host–pathogen interactions is an active research area and has been extensively reviewed (Szempruch *et al*. 2016; Raab-Traub and Dittmer 2017; Freitas *et al*. 2019; Gill, Catchpole and Forterre 2019; de Souza and Barrias 2020; Torrecilhas *et al*. 2020; Rizzo, Rodrigues and Janbon 2020a; Piffer *et al*. 2021; Sabatke *et al*. 2021).

In fungi, ultrastructural analysis of *Aspergillus nidulans* protoplasts carried out early in the 1970s showed EV-like particles pinching off from fungal plasma membrane, then referred to as ‘subprotoplasts’ (Gibson and Peberdy 1972). The presence of secreted vesicles was then indicated in 1973 in the fungal pathogen *Cryptococcus neoformans* (Takeo *et al*. 1973). After these initial microscopical evidences, EV structure and composition were first described more than 30 years later in *C. neoformans* (Rodrigues *et al*. 2007, 2008) and the number of studies characterizing EVs in fungi have been incredibly increasing since then. Fungal EVs have been described in more than 50 independent studies comprising pathogenic and nonpathogenic species, and the timeline concerning most of these reports was recently reviewed (de Oliveira *et al*. 2020; Rizzo, Rodrigues and Janbon 2020a).

Extracellular vesicle production is a relevant mechanism of virulence-associated secretion of molecules. It has been described in most of the primary, opportunistic and emerging fungal pathogens, comprising species from the genera *Candida*, *Cryptococcus*, *Histoplasma*, *Aspergillus*, *Sporothrix*, *Paracoccidiodes*, *Malassezia*, *Rhizopus*, *Exophiala*, *Pichia*, *Trichophyton* and *Talaromyces* (Albuquerque *et al*. 2008; Gehrmann *et al*. 2011; da Silva *et al*. 2015; Gil-Bona *et al*. 2015; Bitencourt *et al*. 2018; Leone *et al*. 2018; Liu *et al*. 2018; Peres da Silva *et al*. 2019; Souza *et al*. 2019; Brauer *et al*. 2020; Karkowska-Kuleta *et al*. 2020; Lavrin *et al*. 2020; Rizzo *et al*. 2020b; Yang *et al*. 2021). EVs were also characterized in many phytopathogens such as *Alternaria infectoria* (Silva *et al*. 2014), *Trichoderma reesei* (de Paula *et al*. 2019), *Fusarium oxysporum f. sp. vasinfectum* (Bleackley, Dawson and Anderson 2019), *Zymoseptoria tritici* (Hill and Solomon 2020) and *Penicillium digitatum* (Costa *et al*. 2021). They have also been described in numerous nonpathogenic species such as *Saccharomyces cerevisiae* (Albuquerque *et al*. 2008), the industrial fungus *T. reesei* (de Paula *et al*. 2019) and different wine yeast species, including *Torulaspora delbrueckii*, *Lachancea thermotolerans*, *Hanseniaspora uvarum*, *Candida sake* and *Metschnikowia pulcherrima* (Mencher *et al*. 2020).

The increasing number of publications highlights the interest of the scientific community in fungal EVs and the relevance of these particles in many contexts, including fungal pathophysiology and host interactions (Rodrigues and Casadevall 2018; Zamith-Miranda *et al*. 2018; de Oliveira *et al*. 2020; Rizzo, Rodrigues and Janbon 2020a; Piffer *et al*. 2021), as well as food biotechnology (Morales *et al*. 2021). However, studies on fungal EV functions are hindered by the limited understanding of their biogenesis, composition and structural diversity. This review explores the recent advances on fungal EVs, focusing on EVs derived from human fungal pathogens.

## EVs IN FUNGI: BIOGENESIS, COMPOSITION, STRUCTURE AND CARGO LOADING REGULATION

### EV biogenesis

Based on the analogy to EV biogenesis in mammalian cells and the analysis of their structure and cargo, it has been hypothesized that fungal EVs could have different cellular sites of origin as reported earlier (Bielska and May 2019; de Oliveira *et al*. 2020; Rizzo, Rodrigues and Janbon 2020a). Electron microscopic approaches revealed that these sites could include the plasma membrane, with events of plasma membrane reshaping and cytoplasmic subtraction (Rodrigues *et al*. 2013) or the direct vesicle shedding (Rizzo, Rodrigues and Janbon 2020a). It was also suggested that EVs could be originated from vesicles-containing vacuoles or via endosome-derived MVB that fuse with the plasma membrane and release EVs to the extracellular space (Rodrigues *et al*. 2008). It was also recently suggested that other organelles, such as the intracellular vesicles clusters (IVCs), are important sites for selecting the EV-associated cargo proteins in *S. cerevisiae* (Winters, Hong-Brown and Chiang 2020).

Despite many reports suggesting potential mechanisms of EV production in fungi, mutant strains that cannot produce EVs are yet to be described, suggesting either functional redundancy of genes and pathways implicated in their formation or essentiality of EV production for membrane biosynthesis and consequently for fungal life (Coelho and Casadevall 2019). Nevertheless, many studies suggest that intracellular trafficking might regulate EV formation. In this regard, some members of the Endosomal Sorting Complex Required for Transport (ESCRT) machinery (Hurley 2015) might be implicated in the control of EV production in fungi, as shown in mammalian cells (Mathieu *et al*. 2019). For instance, Vps2, Vps23, Vps27, Vps36 and Snf7, among others, were shown to regulate the formation, selection of cargo or proper release of EVs in different fungal models such as *S. cerevisiae* (Oliveira *et al*. 2010b, Zhao *et al*. 2019), *Candida albicans* (Zarnowski *et al*. 2018) and *C. neoformans* (Park *et al*. 2020). Accordingly, some of the corresponding mutant strains produced less EVs than the wild type (WT) without being completely deficient in EV production. Similarly, the autophagy regulator Atg7 and the Golgi reassembly stacking (GRASP) were also suggested to affect EV size. GRASP was also reported to be involved in the transport of vesicular mRNA (Peres da Silva *et al*. 2018). Recently, the endocytic adaptor protein Cin1 involved in intracellular vesicular traffic in *Cryptococcus deneoformans* has been shown to regulate EV-mediated RNA release (Liu *et al*. 2020). Also, the anti-fungal drug turbinmicin has been shown to inhibit EV production in *C. albicans* and biofilm formation (Zhao *et al*. 2021). The mechanism of action of turbinmicin is not completely identified. Still, it seems to target a Sec14-dependent intracellular vesicle trafficking pathway, suggesting that the post-Golgi secretory pathways could also be involved in EV production in this yeast (Zhang *et al*. 2020).

Other proteins implicated in lipid metabolism and transport, such as the lipid flippase *APT1* (Rizzo *et al*. 2014) and the lipid scramblase *AIM25* (Reis *et al*. 2019) in *Cryptococcus*, as well as the lipid biosynthetic phosphatidylserine decarboxylase encoding genes, *PSD1* and *PSD2* (Wolf *et al*. 2015) in *C. albicans* were also reported to regulate EV diversity and composition. Similarly, the sterol C-24 methyltransferase encoding gene *ERG6*, involved in ergosterol biogenesis, regulates EV size and cargo in *C. neoformans* (de Oliveira *et al*. 2020). Overall, the above studies suggest that several EV production pathways as well as complex cargo sorting mechanisms exist in fungi.

### EV composition

**Figure 1.**
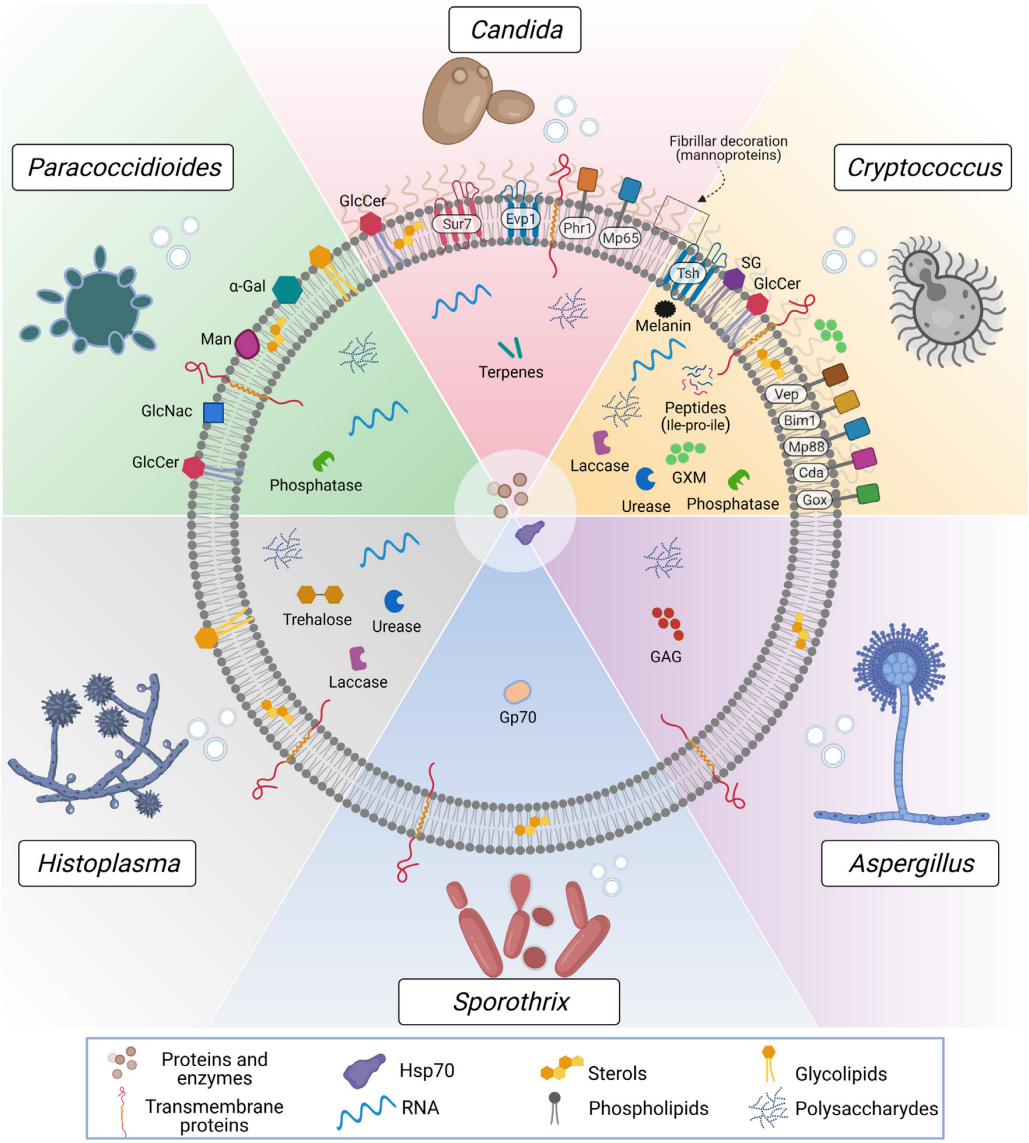
Compositional aspects of EVs released by the main fungal pathogens. EVs are nanoscale lipid bags enclosing a highly complex molecular cargo. EVs have been studied in many pathogenic fungi, including yeast and filamentous species, such as *Cryptococcus*, *Candida*, *Paracoccidioides*, *Histoplasma*, *Sporothrix* and *Aspergillus*. The structure and compositional characterization of fungal EVs are still far from being complete. However, in this scheme, we aimed to summarize the major surface and intraluminal EV components, comprising proteins, polysaccharides, lipids, nucleic acids, small molecules and pigments. Some of the proteins are predicted to be GPI-anchored, such as Phr1 (in *C. albicans*), and Mp88, Vep (Vesicles enriched protein-4 and 5) and other members of the chitin deacetylase family (Cda), the putative glyoxal oxidase family (Gox) and the BCS-inducible membrane protein 1 (Bim1) in *Cryptococcus*. Interestingly, many of these proteins have already been described as highly immunogenic. Other transmembrane proteins, such as Sur7 (*C. albicans*) and Tsh (*Cryptococcus*), are tetraspanins, considered biomarkers of mammalian EVs. Many of those membrane/GPI-anchored proteins are mannosylated and suggested as the molecular composition of the fibrillar decoration on the surface of *C. albicans* and *C. neoformans* EVs revealed by electron microscopy. This mannoprotein-based decoration is not exclusive of pathogenic species since it was also detected in the model yeast *S. cerevisiae*. The presence of this feature needs to be further explored for other pathogens. Carbohydrates involved with stress response were found in *Histoplasma capsulatum* EVs, such as trehalose, and immunogenic polysaccharides were described in fungal EVs, such as the major capsular polysaccharide Glucuronoxylomannan (GXM) in *Cryptococcus* and the exopolysaccharide Galactosaminogalactan (GAG) in *Aspergillus fumigatus*. Glucosyl residues were found on the surface of *Paracoccidiodes* EVs, such as mannose (Man) and N-acetylglucosamine (GlcNAc), together with the immunogenic α-linked galactopyranosyl (α-Gal). Some enzymes were biochemically described as active in fungal EVs, including urease, laccase and phosphatase. Besides the presence of laccase, it has been suggested that its final product, the pigment melanin, was also enclosed in *C. neoformans* EVs. Actually, a model of EV-based melanization has been proposed in *C. neoformans* (Eisenman *et al*. 2009). However, additional experiments are needed to confirm it. Similarly, we cannot exclude the presence of other enzymes detected by proteomics, such as chitinases and cell wall remodeling enzymes, but their activities remain to be confirmed. Some other possible EV-luminal components, such as the 70-kDa glycoprotein (Gp70) in *Sporotrix brasiliensis* were shown to be antigenic, and the peptide leucine-proline-isoleucine (Ile-Pro-Ile) was reported to be biologically active during *C. neoformans in vivo* infection. Among the lipids, many phospholipids, sterols and glycolipids were described, comprising the glycosphingolipid Glucosylceramide (GlcCer) and the sterylglucoside (SG); the latter was detected only in *C. neoformans*. Moreover, terpenes were suggested as the major EVs components inhibiting yeast-to-hyphae transition in *C. albicans*. However, it is unknown if this class of lipids would be associated with membranes or in the lumen of the vesicles. Moreover, although more abundantly found in *C. albicans*, some derivatives were also present in *H. capsulatum* and *S. cerevisiae* EVs. Few other EVs-associated structural lipids were not explored as their biological functions are not deciphered so far. By analyzing the EV-associated proteins among different pathogenic species, it is suggested that the ortholog group of the Heat-shock protein (Hsp70) was the only one found in all these species. For clarity, all the RNA sub-classes identified in fungal EVs were not explored in this figure. The vesicular RNA diversity is detailed in plain text.

Fungal EVs enclose a highly diverse cargo, comprising lipids, proteins, carbohydrates, pigments, nucleic acids, prions, toxins and other small molecules (Rodrigues *et al*. 2007, 2008; Albuquerque *et al*. 2008; Vallejo *et al*. 2012b; da Silva *et al*. 2015; Gil-Bona *et al*. 2015; Kabani and Melki 2015; Vargas *et al*. 2015; Costa *et al*. 2021; Reis *et al*. 2021). However, despite significant advances in new isolation strategies and characterization methods, such as powerful electron microscopy techniques and high-sensitivity nanoparticle analyses (Gandham *et al*. 2020), the characterization of fungal EV composition is still in its infancy. The major components identified in EVs from the most relevant fungal pathogens are described below and summarized in Fig. 1. It is important to highlight that most compositional studies on fungal EVs have not used any enrichment analysis, which can hinder downstream studies. Moreover, although different gradient-based approaches have been suggested to increase the EV purity in mammals (Roux *et al*. 2020), only a few reports using such strategies have been published in fungi (Rodrigues *et al*. 2007; Yang *et al*. 2021). Thus, most published EV components should be considered better as EV-associated until their genuine EV nature could be more carefully addressed. In this sense, a standardization of fungal EV purification protocols is necessary in order to identify fungal EVs markers as well as for compositional and functional analyses.

#### Proteins

Different studies exploring fungal EV proteomics were recently discussed (Bleackley, Dawson and Anderson 2019; de Toledo Martins *et al*. 2019). Overall, proteins related to cell wall biogenesis, plasma membrane, stress responses, pathogenesis, transport and signaling are the most commonly identified in EVs from multiple species (Nimrichter *et al*. 2016; Bleackley, Dawson and Anderson 2019). Notably, the activity of many virulence-associated enzymes was identified in fungal EVs. In *C. neoformans*, laccase, as well as urease and acid phosphatase activities, were described to be associated with EVs (Rodrigues *et al*. 2008). Interestingly, it has been recently shown that Vps27, a key protein of the ESCRT-0 complex essential for virulence in *C. neoformans*, is required for the laccase transport to the cell wall (Park *et al*. 2020). A *vps27Δ* mutant also accumulated MVB and showed a partial defect in EV production, releasing EVs with larger size distribution than wild type, emphasizing the hypothesis that EV-mediated transport of enzymes is an important mechanism for fungal pathogenesis. The presence of active enzymes associated with EVs was also observed in other species, such as a laccase in *Paracoccidoides brasiliensis* (Vallejo *et al*. 2012a), as well as a laccase and an urease in *H. capsulatum* (Baltazar *et al*. 2018). A β-glucosidase was also reported to be associated with EVs in *T. reesei*(de Paula*et al*.^2019^).

A total of two recent EV-protein enrichment analyses suggest the existence of EV-marker proteins in fungi. In *C. albicans*, a list of 22 proteins as EV markers, including the claudin-like Sur7 family proteins (including Sur7 and Evp1) and the GPI-anchored glycosidase Phr1, have been suggested (Dawson *et al*. 2020). In *Cryptococcus*, 17 EV-associated proteins in all three species, *C. neoformans*, *C. deneoformans* and *Cryptococcus deuterogattii* represent potential cryptococcal EV markers (Rizzo *et al*. 2021). Additionally, six membrane-associated protein families were described as typically associated with these EVs, including the chitin deacetylase (Cda) family, the Ricin-type beta-trefoil lectin domain-containing protein (Ril) family, the putative glyoxal oxidase (Gox) family, the tetraspanin membrane proteins containing a SUR7/PalI family motif (Tsh), the pr4/barwin4 domain protein (Blp) family and the multicopper oxidase (Cfo) (Rizzo *et al*. 2021). Interestingly, some of these proteins were described as immunogenic, such as Mp65 in *C. albicans* (Gomez *et al*. 2000) and Mp88 in *C. neoformans* (Levitz *et al*. 2001; Huang *et al*. 2002). Antigenic proteins were also identified for other fungal EVs, such as the 70-kDa glycoprotein (Gp70) in *S. brasiliensis* (Ikeda *et al*. 2018), which was reported as an important virulence factor in *Sporothrix* (de Almeida *et al*. 2015).

Using an orthology approach, a comparative analysis of EV protein content from multiple fungal species has been recently conducted (Parreira *et al*. 2021). The data were compiled in a novel free web repository created for fungal EV datasets, named ExVe (http://exve.icc.fiocruz.br). Interestingly, the OG6_100083 (Hsp70 Pfam domain) was the only ortholog group found in all considered species, suggesting that heat-shock related proteins might play a universal role in fungal EVs (Parreira *et al*. 2021).

#### Lipids

The lipid composition of fungal EVs is still poorly described. The published data are also difficult to compare and integrate due to the diversity of strategies employed in these analyses. Nevertheless, lipid analysis by mass spectrometry revealed that virulence-associated glycosphingolipids, such as glucosylceramide (GlcCer) and sterols are enriched in cryptococcal EVs (Rodrigues *et al*. 2007). Similarly, in *C. albicans* EVs, the major lipids detected by thin-layer chromatography were sterols (ergosterol and lanosterol) and GlcCer (Vargas *et al*. 2015)., Mass spectroscopy identified many phospholipids in *H. capsulatum* EVs (Albuquerque *et al*. 2008). More recently, lipidomic analysis detected on hundred different lipids, comprising phospholipids, triacylglycerides (TG), diacylglycerides (DG), ceramides, sphingomyelins and fatty acids (FA), suggesting a very complex organization of the lipidic membrane of EVs (Cleare *et al*. 2020). Interestingly, the EV lipid profile obtained from cells grown in distinct media was remarkably different (Cleare *et al*. 2020). Similarly, a detailed EV lipidomic analysis performed in *P. brasiliensis* revealed two species of monohexosylceramide and 33 phospholipids, together with glycolipids such as GlcCer, and sterols like brassicasterol, ergosterol and lanosterol (Vallejo *et al*. 2012a).

Thus, the knowledge of the lipid content in fungal EVs appears today as a very long list of lipid classes without any associated function. A better understanding of fungal lipid components is fundamental since many EV-associated lipids can be closely linked to virulence during infection, as for the immunogenic glycolipid sterylglucoside (SG), which seems to be enriched in *C. neoformans* EVs and can induce protection of the invertebrate host *Galleria mellonella* against a lethal challenge with *C. neoformans* (Colombo *et al*. 2019). Similarly, *C. albicans* EVs can inhibit yeast-to-hyphae differentiation, this effect being mediated by EV associated lipids (Honorato *et al*. 2021).

#### Carbohydrates

Approaches to characterize the fungal EV glycol-profile have also been undertaken. In *C. neoformans* immunogold labeling using a specific monoclonal antibody suggested that the presence of the capsular polysaccharide glucuronoxylomannan (GXM) associated with EVs (Rodrigues *et al*. 2007). Similarly, in 2011, enzyme-linked immunosorbent assays (ELISA) revealed the presence of immunogenic α-linked galactopyranosyl (α-Gal) epitopes associated with *P. brasiliensis* EVs (Vallejo *et al*. 2011). Subsequently, *Paracoccidioides* EV fractions were exhibited to contain residues of glucose (Glc), mannose (Man) and galactose (Gal), comprising a high molecular mass α-glucan and a galactofuranosylmannan, which were resolved through high-resolution analyses and determined to dominate the population of EV polysaccharides (da Silva *et al*. 2015). The presence of small amounts of cell wall-associated polysaccharides such as (1-3)-glucan and (1-6)-glucan was also confirmed. Moreover, plant lectin microarray profiling revealed terminal Man and N-acetylglucosamine (GlcNAc) residues exposed at the EV surface (da Silva *et al*. 2015).

It was also suggested that the vesicular polysaccharide content (specifically mannan and glucan) could be a major source of the extracellular polymeric matrix material in *C. albicans* biofilms (Zarnowski *et al*. 2018). In this study, exogenous wild type EVs were able to rescue the matrix production in ESCRT mutant biofilms. In *A. fumigatus*, it was also shown that protoplasts submitted to cell-wall regeneration change their carbohydrate-associated EV profile compared to non-regeneration conditions (Rizzo *et al*. 2020b). Thus, the immunogenic polysaccharide galactosaminogalactan (GAG) was detected in EVs during cell wall synthesis but not in control, revealing that the EV carbohydrate composition changes during fungal cell wall biosynthesis. The amount and diversity of EV-associated carbohydrates is also dependent on the conditions of fungal growth. For instance, EVs obtained from *H. capsulatum* cells grown on Ham's medium presented the highest amount of carbohydrates associated with energy production and storage, whereas trehalose, a stress tolerance related carbohydrate, was more abundant in EVs obtained from cells grown on BHI medium (Cleare *et al*. 2020).

#### Prions, small molecules and mycotoxins

In fungi, the EV metabolome is generally poorly described. Nervertheless, the presence of EV-associated toxins was suggested in few fungal models, such as the cotton pathogen *F. oxysporum f. sp. vasinfectum* (Bleackley, Dawson and Anderson 2019). In the phytopathogen *P. digitatum*, untargeted metabolomics have been identified as EV-metabolite, including tryptoquialanines and the mycotoxin fungisporin, which are important small molecules for plant pathogenesis (Costa *et al*. 2021). By using chromatographic and spectrometric approaches and metabolomic techniques, small molecules were identified in EVs obtained from the pathogen *C. deuterogattii*. One of these vesicular peptides, chemically characterized as leucine-proline-isoleucine (Ile-Pro-Ile), was reported as biologically active during fungal infection in the *G. mellonella* model (Reis *et al*. 2021). These recent studies suggest that EV-associated small molecules could play major roles in host–pathogen interactions and pave the way for further discoveries in this field.

Finally, it has been demonstrated that EVs can also export proteinaceous infectious particles (prions), such as the prion Sup35p (Kabani and Melki 2015). Sup35p is exported in *S. cerevisiae* EVs in its soluble and aggregated infectious states, indicating that prions can also be transmitted between cells through fungal EVs.

#### RNA

For more than a decade, the composition, function and diversity of RNA molecules in EVs has been the focus of a large number of studies in mammals and in some microorganisms. Accordingly, in 2015, a solid sequencing approach was used to identify several EV-associated RNA sub-classes by *C. neoformans*, *P. brasiliensis*, *C. albicans* and *S. cerevisiae* (da Silva *et al*. 2015). They identified mRNAs, microRNA-like (miRNA-like) and several sub-classes of non-coding RNAs (ncRNAs), including transfer RNA (tRNA), ribosomal RNA (rRNA), small nucleolar RNA (snoRNAs) and small nuclear RNA (snRNA)s. Since then, few studies reported additional RNA sub-classes in fungal EVs depending on the employed sequencing strategy. For instance, *Malassezia sympodialis* produces exosome-like vesicles called MalaEx, which carry very small RNAs of unknown function in a size range of 16–20 nucleotides (Rayner *et al*. 2017). In *H. capsulatum*, RNA molecules larger than 200 nucleotides such as mRNAs, rRNAs, tRNAs and snRNAs, and snoRNAs were identified (Alves *et al*. 2019). A group of small RNAs (25–40 nucleotides) was also identified in the same study. These RNAs antisense of several mRNAs encoding proteins of unknown function has been suggested to belong to the miRNA sub-class (Alves *et al*. 2019). More recently, the transcriptome of *C. deneoformans* EVs was characterized, and mRNAs and miRNAs-like associated with these particles were confirmed (Liu *et al*. 2020). This study also identified siRNAs and lncRNAs as the major components of the *C. deneoformans* extracellular transcriptome (Liu *et al*. 2020). Accordingly, several RNA binding proteins (RBPs) including the main components of the RISC complex were identified in the EV proteome from *H. capsulatum* (Alves *et al*. 2019).

Although data sets describing the fungal EV transcriptome are still small, several reports suggest its plasticity in response to environmental cues and genetic mutations, as well as a large diversity between fungal species and even strains. Thus, several differences in the composition and abundances of ncRNAs and mRNAs have been shown between the EV transcriptome of two *P. brasiliensis* strains and the one of *Paracoccidoides lutzii* (Peres da Silva *et al*. 2019). Similarly, among the 124 mRNAs identified in *H. capsulatum* EVs, 93 were enriched in the highly virulent G217B strain and 31 in the less virulent G186AR strain (Alves *et al*. 2019). Very recently, the analysis of *Candida auris* EV transcriptomes in the presence or absence of caspofungin revealed a dynamic composition of EV specific transcripts (Zamith-Miranda *et al*. 2020). Moreover, mutations in *GRASP*, *ATG7* or *CIN1* have been shown to affect the EV-transcriptome in *Cryptococcus*, suggesting a tight control of its composition (Peres da Silva *et al*. 2018; Liu *et al*. 2020).

As for other mammalian cell-derived EVs, identifying diverse RNA sub-classes associated with EVs raises many questions regarding the life cycle of exRNAs in fungi, including the molecular mechanisms underlying their specific loading in EVs and their biological functions (Gruner and McManus 2021). These recent reports also highlight the need for more studies on the characterization of exRNAs associated with different EV subpopulations in fungi.

### EV structure

Despite initial reports describing differences in the morphology of fungal EV populations (Albuquerque *et al*. 2008; Rodrigues *et al*. 2008), the EV structural diversity remained largely unknown until recently. Thus, recent Cryo-electron microscopy (Crypo-EM) analyses revealed that most *Cryptococcus, C. albicans and S. cerevisiae* EV lipid bilayer are superficially covered by a mixture of molecules assuming a fibrillar decoration, mostly composed of membrane-associated mannoproteins (Rizzo *et al*. 2021). Yet, about 10% of the EVs are not decorated. On average, these EVs are smaller, suggesting different biosynthetic pathways (Rizzo *et al*. 2021). In *A. fumigatus*, electron microscopy (EM) analysis suggested the presence of fibril-like structure of unknown composition covering EVs extracted from cells that grow under conditions that stimulate cell wall synthesis, suggesting that EV structural changes might also be associated with the cellular physiological status (Rizzo *et al*. 2020b).

The presence of mannoproteins in EV preparations was also reported in other fungal pathogens (Dawson *et al*. 2020; Karkowska-Kuleta *et al*. 2020). However, in *M. sympodialis*, which is largely devoid of GPI-anchor proteins and many other fungal typical mannoproteins (Gioti *et al*. 2013), EVs exhibit no evidence of decoration on their surface (Johansson *et al*. 2018). This highlights significant structural diversity in fungal EVs. Many of those fungal-EV associated mannoproteins were previously described as immunogenic proteins (Gomez *et al*. 2000; Specht *et al*. 2017), suggesting that these surface decorations might impact host–pathogen interactions and disease development. Glycans are also a common feature for many types of other non-fungal cell-derived EVs (Williams *et al*. 2018) and different structures covering the vesicular surfaces were also revealed by Cryo-EM; for instance, in myxobacteria (Goes *et al*. 2020), virus-infected cells (Yang *et al*. 2020), human cell lines (Noble *et al*. 2020) and EVs obtained from human fluids such as urine (Wang *et al*. 2019). However, the molecular composition and the biological relevance of these structures remain to be explored.

The fungal EVs size ranges from 10 to 1000 nm, but the observed distribution depends on the technique employed for size measurements (Bielska and May 2019; Piffer *et al*. 2021). It is important to note that larger vesicles (>400 nm) and very small EVs (<50 nm) are not well quantified in most Nanoparticle Tracking Analyses (NTA), and small EVs are not easy to detect by most common flow cytometers. Therefore, a combination of complementary techniques providing single EV imaging and single-particle analyzers is recommended to explore the diversity of EV structure (Théry *et al*. 2018). The most reliable technique to estimate EV size is Cryo-EM, with the caveat that it can only be performed on a limited number of samples. In *C. neoformans*, for instance, the combination of high-resolution flow cytometry and Cryo-EM techniques allowed the identification of a prominent population of EVs smaller than 100 nm, contrasting to the previous NTA and DLS analyses (Rizzo *et al*. 2021).

### EV cargo loading regulation

Although the underlying mechanisms of EV composition regulation remain unknown, the differential loading of specific or enriched EV molecules seems to be influenced by numerous conditions, such as the environment and nutrition status, including conditions posed by the host cells. For instance, in the dimorphic fungus *H. capsulatum*, distinct media conditions alters EV characteristics and payloads, such as the sorting of organic molecules (Cleare *et al*. 2020). Similarly, in *C. deneoformans*, nutrient availability interferes with EV size and cargo (Marina *et al*. 2020). It has been shown that EVs obtained from cells grown on nutrient-poor medium have a higher hydrodynamic diameter, increased activity of virulence-associated enzymes and increased amount of capsular polysaccharide GXM, as compared to EVs obtained from cells grown on rich-medium (Marina *et al*. 2020). *H. capsulatum* EV protein load and release are modulated by specific antibody binding to fungal cell surface proteins (Matos Baltazar *et al*. 2016). The binding of these monoclonal antibodies eventually alter EV-associated laccase activity, thereby influencing the pathophysiological roles of these EVs during the host-pathogen interaction (Baltazar *et al*. 2018).

Some other host factors can also affect EV stability. For instance, serum albumin could lead to the disruption of *C. neoformans* EVs (Wolf, Rivera and Casadevall 2012). Moreover, the mammalian β-galactoside-binding protein Galectin-3 (Gal-3), which is increased during cryptococcal infection, was described to inhibit C*. neoformans* growth and exerts a direct lytic effect on fungal EVs, thereby reducing EV stability and possibly changes the course of infection (Almeida *et al*. 2017). Similarly, increasing concentrations of Gal-3 disrupted *P. brasiliensis* EVs in a dose-dependent manner (Hatanaka *et al*. 2019).

## EVs MEDIATING INTERCELLULAR AND INTER-KINGDOM COMMUNICATION PROCESSES

By analogy with their mammalian counterparts, fungal EVs are thought to be a mean for communication *in vivo* and *in vitro* between cells of the same species and with the host during infection. A few examples of EV-based communication in fungi have been reported. In 2018, transwell assays were used to demonstrate that EVs released by *C. deuterogattii* virulent strains can be taken up by infected macrophages where they promote intracellular yeast replication of a less virulent strain. This effect appears to be dependent on some proteins and RNAs, as suggested through proteinase K and RNAse EV treatments (Bielska *et al*. 2018). These results suggest EV-based long-distance pathogen-to-pathogen communication regulating virulence in this fungal pathogen. This hypothesis is enforced by *in vivo* studies, where pre-incubation of poorly virulent *C. neoformans* cells with EVs produced by a hypervirulent strain increases the virulence of the recipient cells when tested in a *G. mellonella* larvae model of infection (Hai *et al*. 2020).

In *C. albicans*, EVs produced by yeast cells can inhibit filamentation and biofilm formation, suggesting that growth conditions and the status of the cells impact the biological properties of EVs in fungi (Honorato *et al*. 2021). This study also reported that yeast cells previously incubated with *C. albicans* EVs, loose their ability to invade the agar and became avirulent in *G. mellonella* larvae (Honorato *et al*. 2021). In contrast, EVs produced from *C. albicans* cells grown under biofilm conditions were shown to complement the extracellular matrix accumulation defect and fluconazole hypersensitivity of ESCRT mutants (Zarnowski *et al*. 2018). These studies suggest different properties and composition of EVs isolated from yeast cells and filaments in *C. abicans*. Interestingly, adding exogenous EVs reduced the efficacy of combined therapy (turbinmicin–fluzonazole) on *C. albicans* biofilms (Zhao *et al*. 2021).

Because EV-associated RNA species play central roles in intercellular communication in other models (Tsatsaronis *et al*. 2018), and because of the wide range of RNA molecules identified in fungal EVs, it is predictable that EV RNAs are among these mediators in fungi. Accordingly, in *P. brasiliensis* EVs, several exonic sRNA-like sequences were identified as mapping to the α-amylase coding gene, which is involved in the synthesis of α- and β-glucans, suggesting a potential role of EVs in the regulation of the expression of these virulence factors (Peres da Silva *et al*. 2019). Moreover, two spectacular examples of fungal extracellular RNAs interacting with the host in a cross-kingdom communication have been reported. The first one describes an extracellular miRNA produced by the entomopathogenic fungus *Beauveria bassiana*, which binds to an argonaute protein of the host *(Anopheles stephensi)* and eventually suppresses the host immunity by silencing the expression of a toll receptor ligand (Cui *et al*. 2019). Yet, it is important to note that the loading of this miRNA in fungal EVs remains to be demonstrated. Similarly, an extracellular miRNA produced by the phytopathogenic fungus *Botrytis cinerae* was shown to hijack the *Arabidopsis* RISC complex, suppressing the host immunity (Weiberg *et al*. 2013). Although the delivery pathway was not identified, it is reminiscent of an EV-based host-pathogen interaction. Interestingly, in that model, EVs produced by the host were shown to contain siRNAs together with several *Arabidopsis* RNA-binding proteins that were able to penetrate fungal cells and inhibit the expression of fungal virulence factors (Cai *et al*. 2018; He *et al*. 2021). Other examples of fungal-plant interaction are from the studies on the cotton pathogen *F. oxysporum f. sp. vasinfectum*, which was shown to release EVs regulating phytotoxic response in the leaves of *Nicotiana benthamiana* (Bleackley, Dawson and Anderson 2019). Recently it has been shown that EVs from *P. digitatum* regulate the germination of *Citrus sinensis* seeds by releasing EV-associated small molecules produced during plant infection, such as the alkaloid tryptoquialanine A (Costa *et al*. 2021).

## MECHANISMS OF EV-BASED TRANSFER OF INFORMATION

The mechanisms by which EVs can transfer information from a donor fungal cell to recipient cells (either fungal or host cells) remain poorly understood. For instance, the model of EV based long-distance communication regulating intracellular replication of *C. deuterogattii* (Bielska *et al*. 2018) implies that EVs cross the cell wall from the donor cells, the membrane of the macrophage, reach the phagolysosome in which these yeasts can replicate, and finally the cell wall and plasma membrane of the recipient yeast cells.

Several hypotheses raise the concern on the mechanisms by which fungal EVs can bidirectionally cross the fungal cell wall (Rodrigues and Casadevall 2018). Nevertheless, liposomes containing the antifungal drug amphotericin B were shown to cross the fungal cell wall, suggesting that EVs could also do the same (Walker *et al*. 2018). Moreover, proteomic analysis of *S. cerevisiae* EVs revealed enrichment in enzymes implicated in cell wall biosynthesis, and EV-based complementation assays suggested potential roles for yeast EVs in the cell wall remodeling. This enrichment of cell wall-related proteins in EV proteome was also observed in other fungi suggesting common properties. Nevertheless, the mechanisms through which EVs can cross the fungal cell wall and plasma membrane remain to be described.

Some experiments using actin polymerization inhibitors or cholesterol depleting agents suggested that fungi EV uptake by the host cell is an active rather than a passive process (Bielska *et al*. 2018). However, the impact of clathrin-mediated or clathrin-independent endocytosis (macropinocytosis and phagocytosis) or the endocytosis via caveolae and lipid rafts for the EV internalization, previously described as the main mechanisms of EV uptake by host cells in other models, remain to be elucidated in fungi. In the last few years, few studies started to explore the mechanisms regulating these processes. Thus, *C. albicans* EVs were shown to co-localize with the plasma membrane ganglioside GM1 of bone marrow-derived macrophages and dendritic cells. Since GM1 is a raft marker, it was suggested that EV internalization might be mediated by lipid rafts. *M. sympodialis* EVs were internalized by both keratinocytes and monocytes at 37°C but not at 4°C,  suggesting that EVs could actively be engulfed by endocytosis. Co-localization assays using *C. neoformans* EVs and FITC-labeled cholera toxin subunit B suggested that fungal EVs are not fusing with the plasma membrane of the macrophages (Oliveira *et al*. 2010a). However, it has been demonstrated that EVs from the same fungal pathogen fuse with human brain microvascular endothelial cells (HBMECs) membranes and potentiate fungal brain invasion (Huang *et al*. 2012). Although mammalian lectin microarray profiling assays suggested that DC-SIGN receptors could recognize surface carbohydrates in *P. brasiliensis* and *P. lutzii* EVs, very little information is available concerning the surface recognition of fungal EVs by the host cells.

## *IN VITRO* AND *IN VIVO* EV-MEDIATED FUNGAL–HOST INTERACTIONS

With the notable exceptions of *Malassezia* spp and *C. albicans*, human fungal infections are mostly due to pathogens living in the environment (Janbon *et al*. 2019). In these cases, the main route of contamination is the respiratory tract, leading to lung infection. Therefore, the first lines of defense against the pulmonary fungal infection are the alveolar macrophages. In the later stages of infection, phagocytes, including monocytes, macrophages, dendritic cells and neutrophils, are involved in nearly all the steps of the host–pathogen interactions. In the past few years, many studies and reviews focused on these immune cells in which fungal pathogens, once internalized, can be either eliminated or survived and multiplied during the infection (Gilbert, Wheeler and May 2014; Heung 2020; Weerasinghe and Traven 2020).

Because of their complex composition, fungal EVs are studied for their impact on the host immune cells. Here again, due to the lack of standardization for fungal EVs purification protocols and to the absence of identified EV specific markers, the following discussed studies need to be regarded as using EV extracts more than purified EVs. Therefore, the associated conclusions should be taken cautiously and might be challenged by experiments using purified EVs in the future. Nevertheless, RAW 264.7 murine macrophage cell line was used to suggest that *C. neoformans* EVs are biologically active and can stimulate immune cells (Oliveira *et al*. 2010a). In this study, *C. neoformans* EVs were shown to be uptaken by macrophages modulating nitric oxide (NO) concentration and pro-inflammatory cytokines TNF-α production. This overall M1 polarization of the EV-treated macrophages was, as expected, associated with an increase in the phagocyte fungicidal activity (Oliveira *et al*. 2010a). Surprisingly, EVs treatment also induces anti-inflammatory cytokine response (IL-10 and TGF-β). This apparent conflicting effect was only partly dependent on the presence of capsular polysaccharide, known to be associated with *C. neoformans* EVs (Rodrigues *et al*. 2007).

*C. albicans* EVs were shown to be internalized by both bone marrow‐derived murine macrophages (BMDM) and dendritic cells, in which they modulate both pro-inflammatory and anti-inflammatory cytokines synthesis as well as NO production, in a dose-dependent manner (Vargas *et al*. 2015). Similar data were recently obtained using *Talaromyces marneffei* EVs (Yang *et al*. 2021). In *M. sympodialis*, EVs were shown to induce both TNF-α and IL-4 cytokines production from peripheral blood mononuclear cells (PBMC), albeit the production of only IL-4 was significantly higher when PBMC from patients suffering from atopic eczema were used as compared to healthy controls (Gehrmann *et al*. 2011).

All these observations suggest that the very complex composition of fungal EVs can result in multiple effects on immune cells, depending on the organism from which EVs are isolated and on the patient's status. For instance, EVs extracted from *H. capsulatum* decrease phagocytosis of this yeast by 35% and no significant increase of NO production was observed upon treating BMDM with EVs (Baltazar *et al*. 2018). In contrast, for some organisms, the effect of EVs appears to be only directed towards a Th1 type response. Thus, in recent years, M1 polarization of macrophages upon incubation with fungal EVs has been reported in *P. brasiliensis* (da Silva *et al*. 2016), *Trichophyton interdigitale* (Bitencourt *et al*. 2018) and *Aspergillus flavus* (Brauer *et al*. 2020). Similarly, in *A. fumigatus*, EVs increase macrophage phagocytosis by more than 10% and strongly induced macrophage pro-inflammatory mediators TNF-α and CCL2 production (Souza *et al*. 2019). A similar effect was reported using bone marrow-derived neutrophils, which are primed by *A. fumigatus* EVs to increase their phagocytic capacity (Souza *et al*. 2019) and on dendritic cells using *S. brasiliensis* EVs (Ikeda *et al*. 2018). As produced by pathogens responsible for skin infection, *M. sympodialis* and *T. interdigitale* EVs interactions with keratinocytes have also been studied (Bitencourt *et al*. 2018; Johansson *et al*. 2018; Vallhov *et al*. 2020). *M. sympodialis* EVs are actively internalized by human keratinocytes, inducing up-regulation of ICAM-1 expression (Johansson *et al*. 2018; Vallhov *et al*. 2020). *T. interdigitale* EVs were shown to induce NO, TNF-α, IL-6, IL-1β and IL-8 from human keratinocyte cell line HaCaT cells in a dose-dependent manner.

The above results suggest that fungal EVs could modulate the innate immune response *in vivo*. This hypothesis was tested using the *G. mellonella* larvae model of fungal infection. In agreement with the variable effect of fungal EVs on phagocytes, the outcomes of these assays are diverse. A pre-treatment of *G. mellonella* larvae with *C. albicans* EVs previously stored at –80, –20 or 4°C reduces the fungal burden in this model without statistically significant increase of the larvae survival (Vargas *et al*. 2015, 2020). Strikingly, the same experiment performed with freshly prepared EVs prolonged larvae survival (Vargas *et al*. 2020). In *C. neoformans*, EVs isolated from an acapsular mutant strain does not protect the infected larvae, whereas wild type EVs accelerated their death (Colombo *et al*. 2019). In contrast, prior stimulation of *G. mellonella* larvae with *A. flavus* EVs promoted decreased fungal burden and increased survival of the larvae (Brauer *et al*. 2020). These results further illustrate the complexity of the message carried by fungal EVs.

## PERSPECTIVES—EV-BASED VACCINE FOR FUNGAL INFECTIONS

In bacteria and parasites, EVs have been shown to stimulate adaptive immunity, and several vaccine candidates have been tested in the murine model of infection and clinical assays (Choi *et al*. 2015; Shears *et al*. 2018; Launay *et al*. 2019). In bacteria, EVs have been used for vaccine development since the end of the 1980s, and several EV-based vaccines are already licensed against meningococcal B disease (Acevedo *et al*. 2014). Several observations suggest that fungal EVs could also modulate adaptive immune response (Silverman *et al*. 2010; Pathirana and Kaparakis-Liaskos 2016). For instance, DC exposed to *C. albicans* EVs produce a higher level of CD86 and MHC-II compared to the untreated control (Vargas *et al*. 2015). Similar data recently obtained using macrophages incubated with *T. marneffei* EVs, suggest that EVs could be involved in T cell activation (Yang *et al*. 2021).

The analysis of EV proteome isolated from different species also revealed major known antigens associated with these vesicles. For instance, MP65 is one of the most prominent *C. albicans* EV protein and a major antigen in this pathogenic yeast (Gomez *et al*. 2000; Vargas *et al*. 2015). Similarly, *M. sympodialis* EVs are enriched in the two allergens Mala s 1 and s 7 (Zargari *et al*. 1994; Johansson *et al*. 2018). Also, in *C. neoformans*, EV proteome analysis revealed a spectacular enrichment of several major antigens such as the mannoproteins MP88 and MP98 (Levitz *et al*. 2001; Huang *et al*. 2002; Rizzo *et al*. 2021). Similar results were obtained in *A. fumigatus* and *S. brasiliensis*, suggesting that EV proteins can be antigenic and sensitize the host immune system (Ikeda *et al*. 2018; Souza *et al*. 2019).

Mice pre-treated using *C. albicans* EVs were demonstrated to fully protect against *C. albicans*, whereas non-vaccinated mice died after two weeks (Vargas *et al*. 2020). Immunization was shown to induce specific profiles of cytokines and is associated with a reduced fungal burden in different organs (Vargas *et al*. 2020). Similar data were obtained in *C. neoformans*, although pre-treatment with EVs only prolonged the survival of the mice (Rizzo *et al*. 2021). Interestingly, mice vaccinated with EVs extracted from an acapsular mutant survived longer than the ones vaccinated using wild type EVs, suggesting that the structure and composition of the EVs can also impact their vaccination properties (Rizzo *et al*. 2021).

In contrast, for some species under some conditions, pre-treatment with EVs appeared to be harmful for the disease outcome in mouse model of infection. Thus, in a mouse model of sporotrichosis, skin lesions and fungal burden are enhanced by a pre-subcutaneous injection of EVs (Ikeda *et al*. 2018). More surprisingly, some data suggested that EVs could favor the passage of *C. neoformans* through the blood-brain barrier and thus the virulence of this yeast (Huang *et al*. 2012). This paradoxical impact of fungal EVs during the infection remains poorly understood (Piffer *et al*. 2021) even though it is predictable that they are produced and can interact with the host cells *in vivo*.

## CONCLUDING REMARKS AND PERSPECTIVES ON FUNGAL EV RESEARCH

**Figure 2.**
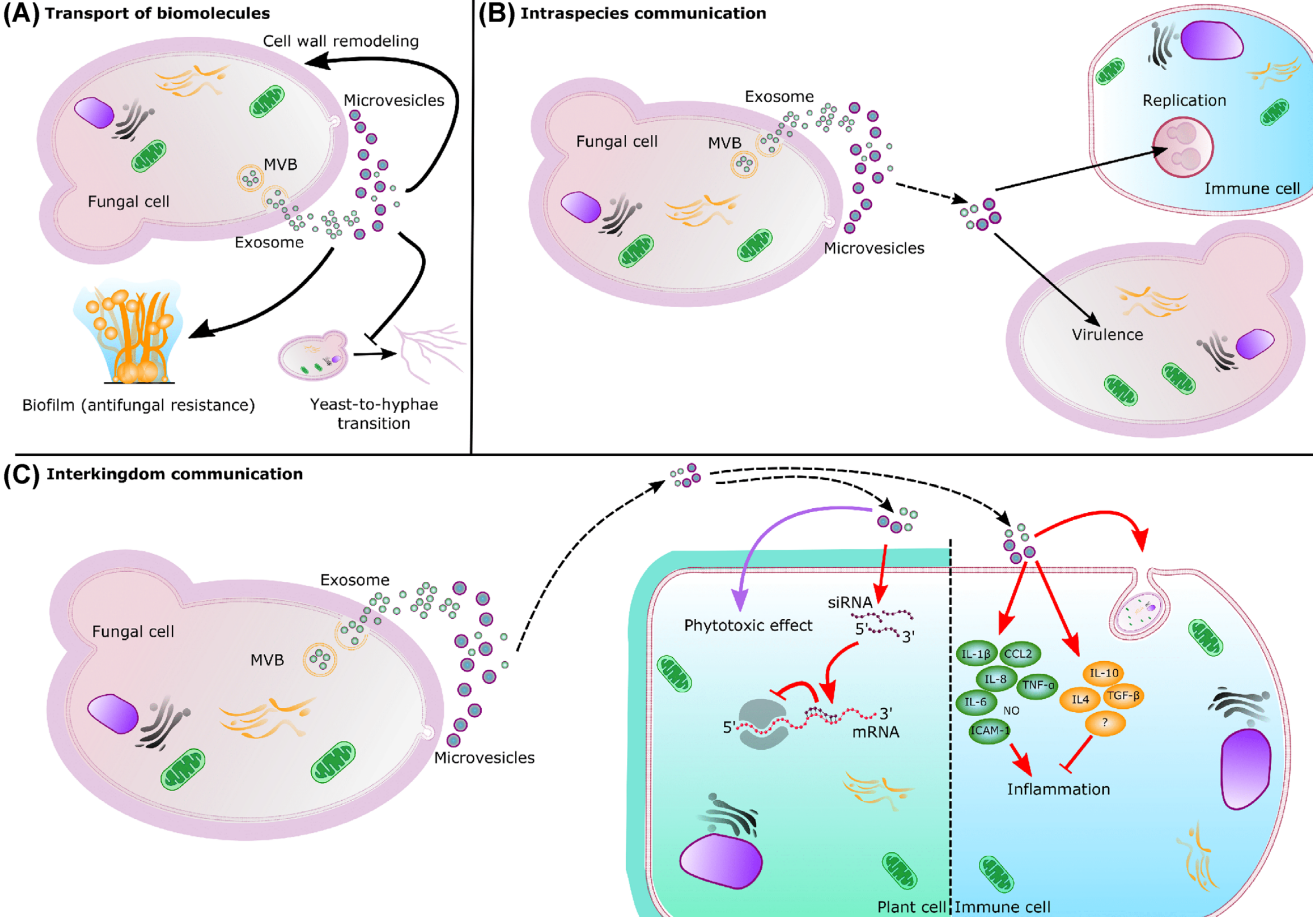
Biosynthesis and roles of fungal EVs. EVs are transported across the cell wall to the extracellular space. Exosomes result from the fusion of multi-vesicular bodies (MVB) with the plasma membrane and microvesicles bud out from the plasma membrane. **(A)** Transport of biomolecules. EVs can carry and release several biomolecules. In *S. cerevisiae*, the enrichment of several cell wall related enzymes in EVs suggests an impact on cell wall remodeling. *In C. albicans*, EVs are able to promote biofilm formation depending on their origin. In other hand, yeast-derived EVs inhibit yeast-to-hyphae differentiation. **(B)** Intraspecies communication. EVs released by *Cryptococcus* donor cells (dashed arrow) can also regulate intracellular replication and virulence of receptor cells. **(C)** Interkingdom communication. Fungal EVs are also implicated in interkingdom interactions influencing immune response (red arrow) during the infection. Fungal siRNAs can enter the plant cells though EVs where their association with the host Argonaute protein inhibits the immune response. Fungal EVs can also have phytotoxic effect (purple arrow) carrying small molecules. In immune cell, fungal EVs can promote the phagocytosis and regulate anti-inflammatory and pro-inflammatory (NO: nitric oxide) responses.

The field of fungal EVs is only in the early stage, and the knowledge basis regarding their structure, composition and function needs to be improved. Although a general picture of the fungal EV starts to be revealed, it remains unclear due to several challenging points. First, the diversity of fungal species studied for EVs, and the expected diversity of fungal EVs render a general comparison complicated. In this context, the creation of other public databases, similar to the ExVe (Parreira *et al*. 2021), designed for fungal EV-proteins that could list and compare fungal EV components among different species, would be instrumental. Second, as it has been done for mammalian EVs, the efforts for standardization of purification and characterization protocols of fungal EVs need to be undertaken aiming to identify specific markers to validate the purity of the EV extracts. Large sets of data related to fungal EVs will undoubtedly be produced soon, but standardized methods would be fundamental for a better knowledge of fungal EV structure and composition. This will also greatly impact the study of the fungal EV functionality. Indeed, the biological function of fungal EVs and their involvement in fungal infections remain obscure. Fungal EVs are expected to participate in many aspects of cell-to-cell communication and host-pathogen interactions. Still, only a few examples of EV functional roles have been published, as discussed in this review and summarized in Fig. 2. Yet, the perspectives are huge, although optimized tools and functional assays remain to be set up. Similar to other EV fields, the diversity of fungal EVs is still poorly studied. For instance, it is known that fungal EVs have different sizes and decorations, but the analysis of the heterogeneity of their cargo has not been fully addressed.

Single EV analyses have started to be implemented for mammalian EVs (Chiang and Chen 2019). This type of analysis demands specific markers and adapted protocols. It is expected to see the fungal EV field following this path as well soon. Similarly, the biosynthetic pathways regulating fungal EVs are also mostly unknown, although few analyses suggest a link between intracellular trafficking and the release of EVs. The existence of microvesicles and exosomes has been hypothesized in fungi by analogy with the mammalian EVs. Indeed, several microscopic and flow cytometry observations tend to support this model. However, the genetic and the mechanisms regulating their production are still underexplored.

Fungi are very well suited to genetic screens. Together with adapted biochemistry and cell biology assays, they might assist in deciphering the mechanisms by which EVs are produced and how they transmit a message to another cell and how this message is read. Additionally, the strength of fungal genetics applied to EV research should help, in some instances, to propose models for metazoan EVs.

Several EV-based vaccines have already been licensed in bacteria. Accordingly, fungal EVs have been considered as vaccine candidates in several studies. It is promising, as no antifungal vaccine yet to be licensed. Fungi are usually very easy to grow and, most of the times, easy to amenable to genetic approaches. Adapted protocols are now accessible to produce fungal EVs on large scales and at a low cost. Specific modification of EVs to improve their vaccine capacities should thus be possible soon and pave the way for developing antifungal vaccines. Overall, the study of EVs in the biology of fungal pathogens and the pathophysiology of fungal infections are evolving fast, which might deeply change the scientific approaches in this field soon.
